# Analysis of radioiodine therapy and prognostic factors of differentiated thyroid cancer patients with pulmonary metastasis: An 8-year retrospective study

**DOI:** 10.1097/MD.0000000000006809

**Published:** 2017-05-12

**Authors:** Renfei Wang, Yueqian Zhang, Jian Tan, Guizhi Zhang, Ruiguo Zhang, Wei Zheng, Yajing He

**Affiliations:** Department of Nuclear Medicine, Tianjin Medical University General Hospital, Anshan Road, Tianjin, People's Republic of China.

**Keywords:** differentiated thyroid cancer, pulmonary metastasis, radioiodine therapy

## Abstract

To assess the efficacy of radioiodine therapy (RIT) and investigate the prognostic factors for patients with pulmonary metastasis secondary to differentiated thyroid carcinoma (DTC) through a retrospective study. A total of 80 patients with radioactive iodine-131 (^131^I)-avid pulmonary metastasis from DTC treated with ^131^I from 2007 to 2014 at our institution entered the study. Treatment response was mainly measured by two parameters: serum thyroglobulin (Tg) levels and post-therapeutic ^131^I whole-body scan (WBS). Treatment variables were assessed for statistical significance using the univariate and multivariate analyses. A receiver-operating characteristic (ROC) curve was also plotted to verify the accuracy of predictors. Of these 80 patients, the overall effective rate was 72.5% (58/80), the rates for complete response (CR), partial response (PR), and no response (NR) were 20.0%, 52.5%, and 27.5%, respectively. Univariate analysis showed that gender, pulmonary nodule size, absence or presence of extrapulmonary distant metastases, age, and Tg level at diagnosis were significantly associated with ^131^I therapy efficacy. Binary logistic regression analysis revealed that older patients (odds ratio [OR]:1.481, 95% confidence interval [CI]: 1.457–2.091, *P* = .020), subjects with higher Tg levels at diagnosis (OR: 1.046, 95% CI: 1.016–1.119, *P* = .014), and those with extrapulmonary distant metastases (OR: 1.185, 95%CI: 1.025–1.463, *P* = .020) had a higher probability of poor prognosis. The optimal cutoffs for age and Tg level to predict ^131^I therapy efficacy for DTC with lung metastases were 46 years old and 55.50 ng/mL, respectively, based on ROC analysis. This study indicated that most DTC patients with pulmonary metastases can obtain partial or complete remission after RIT, while older patients with higher Tg levels at diagnosis and extrapulmonary distant metastases more likely show poor prognosis.

## 1 Introduction

Differentiated thyroid carcinoma (DTC) is one of the most commonly observed types of endocrine cancer. In general, the prognosis of DTC is positive, however, patients exhibiting distant metastases demonstrate a markedly worse prognosis.^[1]^ Lungs are the most frequent distant localization of metastases from DTC, with an incidence rate of 2% to 20%.^[2]^ About 50% of patients with such metastases die within 10 years.^[3]^ Pulmonary metastases are classified as radioactive iodine-131 (^131^I)-avid and non-^131^I-avid. ^131^I could be effective only in metastatic patients with positive ^131^I uptake, and there is no obvious benefit for those with non-^131^I-avid metastases. This analysis only enrolled ^131^I-avid DTC patients with lung metastases.

Radioiodine therapy (RIT) is suitable for those ^131^I-avid but surgically unresectable lung metastases of DTC and can alleviate the condition partially or remove the lesion effectively. It has already become the most crucial means for treating lung metastases of DTC. To date, a few previous investigations have discussed the efficacy of RIT and its influential factors for DTC patients with lung metastases.^[4–9]^ However, there is a large difference on the cure rate and efficacy of DTC patients with lung metastases by RIT in many reports,^[10]^ and the exact factors that influence the efficacy remain uncertain.

The present study is a retrospective chart review of 80 DTC patients with lung metastasis treated and managed at our institution. The aim of this study was to evaluate the therapeutic effects of RIT on DTC patients with pulmonary metastases by serum thyroglobulin (Tg) level at diagnosis and posttherapeutic ^131^I whole-body scan (WBS), filter out the relevant factors associated with therapeutic efficacy of RIT.

## 2 Materials and methods

### 2.1 Patients

A total of 1028 patients received RIT for DTC at our institution between January 2007 and December 2014, and 92 (8.95%) patients were diagnosed with lung metastasis. Among them, 11 patients who had non-^131^I-avid pulmonary metastases and 1 patient who did not receive any further regular follow-ups were excluded; therefore, 80 patients were finally enrolled in the analysis. Among these 80 patients, 52 were female and 28 were male (F:M = 1.86:1). The study was approved by the Institutional Review Board and Ethics Committee of Tianjin Medical University. Written informed consent was obtained from all patients.

### 2.2 Diagnostic criteria for DTC pulmonary metastases

The diagnosis of pulmonary metastases was based on cytological or pathologic confirmation, diagnostic or therapeutic ^131^I-WBS, clinical and postoperative, or follow-up other imaging techniques. A lung ^131^I uptake value higher than the normal basal level, excluding the physiological uptake and contamination from the body surface, was considered to be WBS-positive. A patient met one of the following criteria was considered to have pulmonary metastases and be included by this study: pathological results confirmed; patients diagnosed with lung metastases of DTC by posttherapeutic or diagnostic ^131^I-WBS after successful remnant ablation; patients diagnosed with lung metastases of DTC by chest X-ray, computed tomography (CT), positron emission tomography-CT, and other imaging techniques.

### 2.3 Procedures for radioiodine therapy

All patients underwent total thyroidectomy, 68 patients (85.0%) also received a lymph node dissection (include central neck lymph node dissection and/or selective lateral neck lymph node dissection). They were given a high-fixed ablative dose of ^131^I therapy 4 to 6 weeks after initial surgery. Before treatment with high-dose radioactive iodine, we will risk stratify our patients in terms of size and number of tumor tissue, histopathology, absence or presence of metastases, type of thyroidectomy, etc. Pretreatment required an iodine-free diet and thyroid hormone withdrawal for 3 to 4 weeks. All patients exhibited a clinical hypothyroid state, with serum thyroid-stimulating hormone levels (TSH) >30 μIU/mL. Conventional measurements, including free tri-iodothyronine, free thyroxine, TSH, Tg, Tg antibody (TgAb), neck ultrasonography, and chest CT scan were performed before ^131^I administration. Tg and TgAb were measured by immunometric assays (Immulite 2000; Siemens Medical Solutions Diagnostics, Los Angeles, CA), and the given normal range was 0 to 55 ng/mL and 0 to 40 IU/mL, respectively. Each patient received 1.85 to 3.70 GBq and 5.55 to 7.40 GBq ^131^I for thyroid remnant and lung metastatic sites, respectively. Levothyroxine (L-T_4_) therapy was replaced 72 hours later, and posttherapeutic ^131^I-WBS was carried out 4 to 6 days after ^131^I administration.^131^I imaging was performed using a dual-detector single-photon emission computed tomography instrument equipped with high-energy parallel-hole collimators (Discovery VH; GE Healthcare, Milwaukee, WI), and interpreted by 2 experienced nuclear medicine physicians. For most patients with ^131^I-avid lung metastases on ^131^I-WBS, RIT was repeated at 6 months and once a year until no further uptake was visible. Periodic follow-up WBS was administrated for patients with normal ^131^I WBS, at first once a year, then every 2 or 3 years, and suppressive therapy was resumed. Additional diagnostic procedures consisted of routine clinical examination, chest CT, neck ultrasonography, serum TSH, and Tg measurements. RIT was discontinued in patients with non-^131^I-avid lung metastases after residual thyroid tissue was completely ablated.

### 2.4 Assessment of therapeutic efficacy

^131^I treatment response was mainly measured by 2 parameters: Tg levels and posttherapeutic ^131^I WBS, supplemented by other imaging studies. The following criteria were used to assess the therapeutic efficacy: complete response (CR), no clinical symptoms of lung metastases, no abnormal lung uptake in ^131^I-WBS and other imaging examinations, and negative Tg (serum Tg levels <1 ng/mL with TSH stimulation or undetectable Tg levels with TSH non-stimulation); partial response (PR), decreased Tg levels, with decreased lung uptake or the volume or number of lung metastasis lesions in ^131^I-WBS, and/or other imaging studies; and no response (NR), increased or no obvious decreased Tg levels, with no change or even deteriorated (higher uptake, increased volume, or number of lung metastasis lesions) in ^131^I-WBS and/or other imaging examinations. For patients with increased Tg levels, lower or even no obvious uptake of lung metastasis lesions in ^131^I-WBS, chest CT must be taken into consideration to assess the therapeutic efficacy. CR and PR were considered to indicate effective ^131^I therapy. The response rates of CR, PR, and NR were analyzed according to the above criteria.

### 2.5 Observation factors

The following 10 factors may be associated with the curative effects of RIT for lung metastases of DTC: age at diagnosis, gender, type of thyroidectomy, histology, pattern of lung uptake in WBS, pulmonary nodule size, cervical nodal metastases, absence or presence of extrapulmonary distant metastases, such as bone or other organ metastases, etc., time of lung metastases, and serum Tg levels at diagnosis. According to results of the chest CT, patients were divided into 3 categories: negative finding, included patients with negative chest CT but positive ^131^I uptake on WBS; pulmonary nodule size ≤1 cm, included patients with all nodules ≤1 cm in diameter measured by CT; and pulmonary nodule size >1 cm, included patients with at least 1 nodule >1 cm in diameter measured by CT.

### 2.6 Statistical analysis

Data are expressed as mean ± standard deviation, proportions, or absolute numbers. All the factors that may have affected the efficacy of ^131^I therapy for patients with pulmonary metastases were analyzed by univariate analysis, performed by Student *t* test and a chi square test. Multivariate analysis, binary logistic regression was used to identify prognostic factors associated with the outcome of ^131^I therapy. Receiver-operating characteristic (ROC) curves were plotted to verify the accuracy for the prediction of ^131^I therapy efficacy for DTC with lung metastases. The area under curve (AUC) was used as an estimation of diagnostic accuracy. All statistical analyses were performed using SPSS version 17.0. All *P* values presented were 2-tailed, and values <0.05 were considered to be statistically significant.

## 3 Results

### 3.1 Characteristics of patients with lung metastases

Among the 80 patients, 68 (85.0%) had papillary thyroid carcinoma and 12 (15.0%) had follicular thyroid carcinoma. The mean age at diagnosis for the primary cancer was 39.61 ± 10.25 years (range, 15–68 years), and the mean follow-up period was 50.21 ± 38.61 months. Additionally, 12 of the 80 patients (15.0%) underwent total thyroidectomy, and 68 patients (85.0%) also received a lymph node dissection. The pulmonary metastases were detected in 62 patients (77.5%) before ^131^I therapy and the remaining 18 patients (22.5%) were diagnosed by WBS during ^131^I therapy.

### 3.2 Evaluation of the efficacy of ^131^I treatment

The following response rates were observed according to the mentioned criteria among the 80 patients investigated: CR, 20.0% (16/80); PR, 52.5% (42/80); and NR, 27.5% (22/80). The overall effectiveness rate was 72.5% (58/80). The mean cumulative radioiodine dose was 20.35 GBq with a range of 8.14 to 50.69 GBq. All patients were administered ^131^I treatment 2 to 9 times, and the average was about 4.1 times. The mean accumulated dosage for the patients who achieved CR, PR, and NR was 14.80 ± 0.65 GBq (range, 8.14–36.26 GBq), 18.87 ± 1.05 GBq (range, 8.51–50.69 GBq), 19.98 ± 1.14 GBq (range, 9.25–32.93 GBq), respectively (*P* = .632). At our last follow-up, all patients were alive. Among the 22 NR patients, 15 patients with increased Tg levels, 10 patients had higher uptake, increased volume or number of lung metastasis lesions in ^131^I-WBS, 6 patients with higher uptake, increased volume or number of lung metastasis lesions in ^131^I-WBS and chest CT, and 4 patients had lower or even no obvious uptake of lung metastasis lesions in ^131^I-WBS, while deteriorated in chest CT.

### 3.3 Univariate analyses for the prognostic factors of ^131^I therapy efficacy for DTC with lung metastases

The results of the univariate analyses that may influence the efficacy of ^131^I therapy for lung metastases of DTC are given in Tables 1–2. The results showed that older patients, males, cases with higher stimulated Tg levels at diagnosis, pulmonary-nodule size greater than 1 cm, and those with extrapulmonary distant metastases more likely had poor prognosis (*P* *=* .019, .026, .012, .039, and .019, respectively). However, we found no statistically significant differences in the type of thyroidectomy (*P* = .855), pathological type (*P* = .754), with or without cervical nodal metastases (*P* = .762), time of lung metastasis at diagnosis (*P* = .721), and pattern of lung uptake in WBS (*P* = .810).

**Table 1:**
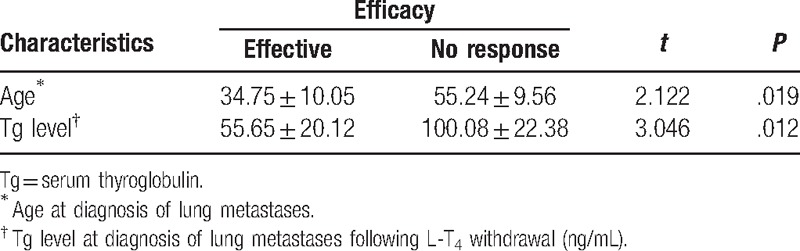
Univariate analyses for the continuous variables.

**Table 2:**
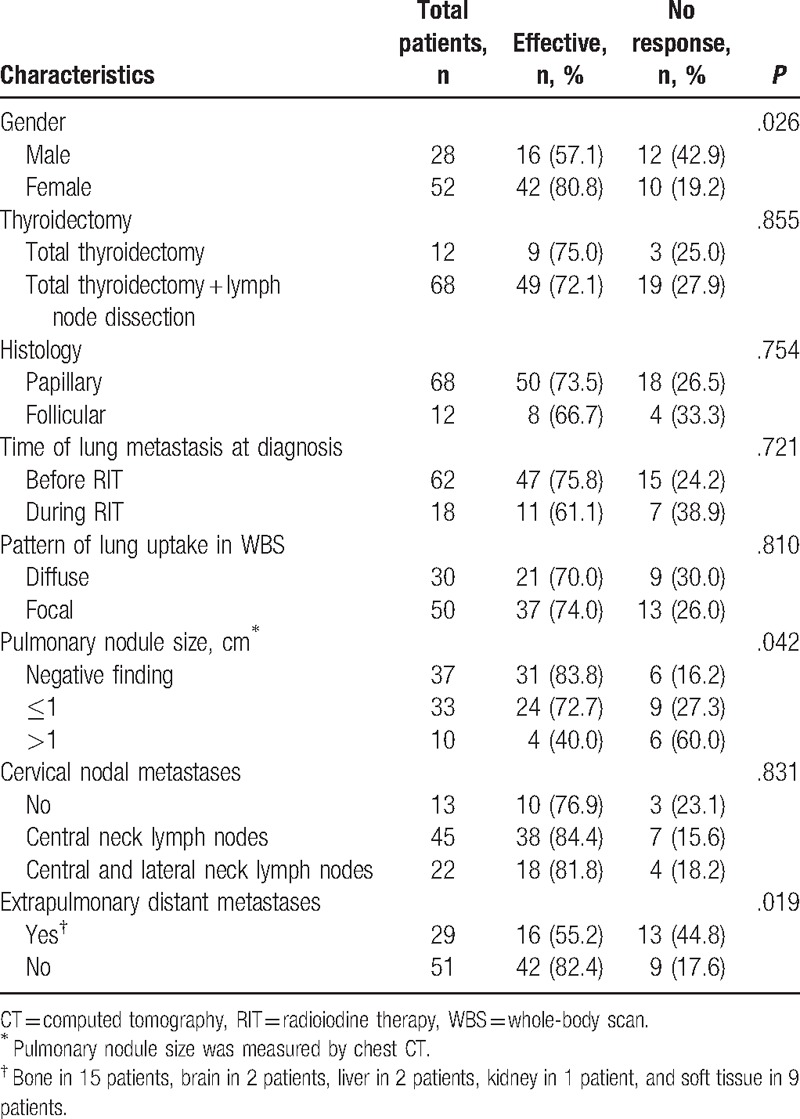
Univariate analyses for the categorical variables.

### 3.4 Multivariate analyses for the prognostic factors of ^131^I therapy efficacy for DTC with lung metastases

Table 3 shows a multivariate analysis of the influential factors of ^131^I therapy efficacy for DTC with lung metastases. Variables that were significant in the univariate analysis were entered into the binary logistic regression analysis using a stepwise method. The results revealed that age, serum Tg levels at diagnosis, and absence or presence of extrapulmonary distant metastases were the independent factors predicting ^131^I therapy efficacy for DTC with lung metastases. Furthermore, we found older cases (odds ratio [OR]: 1.481, 95% confidence interval [CI]: 1.457–2.091), subjects with higher Tg levels at diagnosis (OR: 1.046, 95%CI: 1.016–1.119), and those with extrapulmonary distant metastases (OR: 1.185, 95%CI: 1.025–1.463) had a higher probability of NR (poor prognosis). Then, we can get the regression equation: Logit P = **−**2.716 + 0.049age + 0.236Tg level + 1.025 extrapulmonary distant metastasis.

**Table 3:**
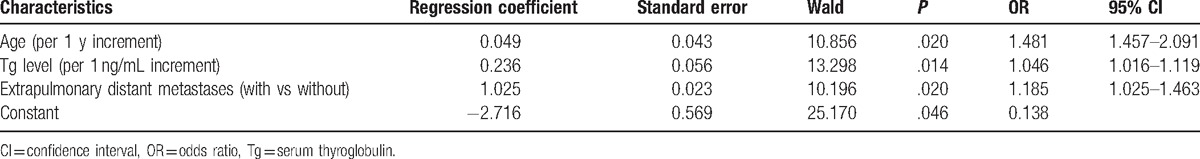
Multivariate analyses for the variables using binary logistic regression.

### 3.5 ROC curves of the prognostic factors of ^131^I therapy efficacy for DTC with lung metastases

**Figure 1:**
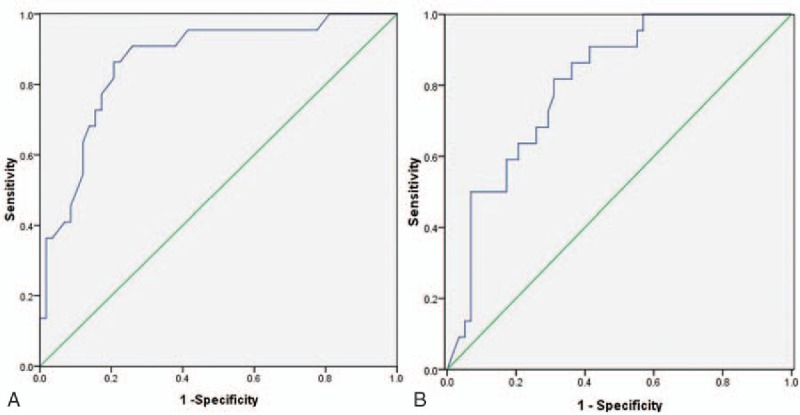
ROC curves for age (A) and Tg level (B) in predicting ^131^I therapy efficacy for DTC with lung metastases. DTC = differentiated thyroid carcinoma, ^131^I = radioactive iodine-131, ROC = receiver-operating characteristic, Tg = serum thyroglobulin.

ROC curves were drawn to evaluate the accuracy of age and Tg level in predicting ^131^I therapy efficacy for DTC with lung metastases (Fig. 1). From the ROC curves we can obtain the optimal cutoff values yielding maximum sums of sensitivity and specificity.^[11]^ The results demonstrated that the optimal cutoff value for age was 46 years old, at which the sensitivity and specificity were 86.4% and 79.3%, respectively (AUC: 0.866; 95% CI: 0.777–0.955, *P* < .001). The optimal cutoff value for Tg was 55.50 ng/mL, at which the sensitivity and specificity were 81.8% and 69.0%, respectively (AUC: 0.807; 95% CI: 0.710–0.904, *P* < .001).

## 4 Discussion

In this series of patients with ^131^I-avid thyroid cancer metastatic to the lungs, we assessed the efficacy of ^131^I therapy and investigated the prognostic factors. The current study found that 3 variables, age, serum Tg level at diagnosis, and the absence or presence of extrapulmonary distant metastases were independent significant predictors of the efficacy of ^131^I therapy.

Tg level and posttherapeutic ^131^I WBS were used to measure the treatment response of RIT in this study. Consistent with previous studies,^[4,12]^ the effective and CR rates of RIT for DTC patients with lung metastases in this study were 72.5% and 20.0%, respectively. ^131^I imaging and Tg level analysis played important role in evaluating the efficacy of RIT for DTC with lung metastases, and comprehensive judgments should be made in combination with the patient's general conditions.^[13]^ The reasons for the clinical outcome of Tg-positive, WBS-negative patients may be as follows: the tumor dedifferentiated and became refractory to radioactive iodine; the recurrent tumor was too small and was below the sensitivity of ^131^I scanning; or there was a dissociation between Tg synthesis and the iodine-trapping mechanism.^[14,15]^ For the clinical outcome of Tg-negative, WBS-positive patients, there are some possible explanations: the serum TgAb caused some negative interference in the measurement of Tg by the electrochemiluminescence immunoassay, which affected the accuracy of measuring the Tg levels^[16,17]^; the tumor synthesized aberrant Tg or could not synthesize and secrete Tg^[18]^; or the ^131^I scan provided false positive results. Only when Tg and ^131^I scan were both negative and no other structural or functional evidence of disease was observed, could it be determined that an excellent response had been achieved (remission, no evidence of disease). When Tg and ^131^I scan were both positive, well-differentiated DTC metastases could be treated with ^131^I. ^131^I refractory status should be considered and the pros and cons of RIT should be reassessed for those DTC patients with a poor curative effect, without obvious fading of lesions, or tumor progression.^[19]^ RIT should be abandoned in favor of other treatment modalities, such as molecular targeted therapy or external beam radiotherapy for those lesions that lose the ability to concentrate radioiodine because of a loss of differentiation or other causes.^[20,21]^

The efficacy of ^131^I therapy for lung metastases of DTC is influenced by many factors. In this study, univariate analysis showed that gender, pulmonary nodule size, age, Tg level at diagnosis, and the absence or presence of extrapulmonary distant metastases were associated significantly with ^131^I therapy efficacy. Furthermore, multivariate logistic regression analysis indicated that age, serum Tg level at diagnosis, and the absence or presence of extrapulmonary distant metastases were independent influential factors for ^131^I therapy efficacy. Using 46 years old as a cutoff, for the prediction of ^131^I therapy efficacy for DTC with lung metastases, the sensitivity and specificity of age were 86.4% and 79.3%, respectively, and the sensitivity and specificity of Tg level using 55.50 ng/mL as cut-off were 81.8% and 69.0%, respectively.

Age is known to be an independent prognostic factor for the efficacy of ^131^I therapy in DTC.^[8,9,22,23]^ The present study showed that age could predict the efficacy of RIT for DTC patients with lung metastases. Younger patients had better responses to RIT than older patients. Chopra et al, in a study of 200 Indian DTC patients with pulmonary metastases, found that patients’ age was an independent prognostic factor predicting disease remission after ^131^I therapy.^[7]^ In a retrospective study conducted from 1962 to 2009 in Korea, which included 152 DTC patients with lung metastases, Cho et al^[24]^ reported that poor prognosis was associated more frequently with older age. Consistent with these studies, the present study showed a lower effective rate after RIT in older patients, confirming that older age was related to lower efficacy of ^131^I therapy. Therefore, we concluded that the underlying mechanisms that might explain the association between older age and poor ^131^I therapy efficacy included a relatively longer disease duration, lower radiation sensitivity, a decline in the immune system, a more aggressive variant of thyroid carcinoma, and a more advanced stage of the illness.^[9,25,26]^ Collectively, we deduced that although most DTC patients with pulmonary metastases can obtain partial or complete remission after RIT, all metastases should be treated at an early stage.

Numerous studies have shown that the serum Tg level at diagnosis is an independent prognostic indicator for ^131^I therapy efficacy.^[17,27]^ Song et al,^[4]^ in a retrospective study of 372 Chinese DTC patients with pulmonary metastases, found that the serum Tg level was a prognostic indicator to assess the efficacy of ^131^I therapy for pulmonary metastases. In agreement with the previous studies, the current research also found that patients with higher Tg levels at diagnosis were more likely show poor prognosis. Serum Tg is produced exclusively by the thyroid gland; therefore, the measurement of serum Tg levels is an important modality to monitor patients for residual or recurrent disease.^[28–30]^ We suggested that DTC patients with lung metastases and high Tg levels at diagnosis might have with more metastatic tissues that could synthesis and secrete Tg. However, there was no obvious relationship between synthesis of Tg and iodine uptake for the metastatic tissues. Patients with high levels of Tg might have less ^131^I-avid lesions. These might explain why the efficacy of ^131^I therapy was worse in some patients with high Tg levels in the present study.

In addition, our study showed the presence of extrapulmonary distant metastases could predict a poor prognosis for RIT. This agreed with a cohort study of 52 patients with DTC and distant metastases conducted in America by Nixon et al,^[31]^ which indicated that the presence of extrapulmonary metastases was a significant predictor of the poor outcome, despite thyroid surgery and RIT. In the present study, 29 patients had extrapulmonary distant metastases: in the bone in 15 patients, in the brain in 2 patients, in the liver in 2 patients, in the kidney in 1 patient, and in soft tissue in 9 patients. Poor prognosis associated with extrapulmonary distant metastases could be explained as follows. The patients with concomitant extrapulmonary distant metastases had an additional site of metastases compared with those with pulmonary lesions alone. Previous studies have shown that multiple sites of metastases are associated with particularly poor prognosis.^[4,7]^ Second, bone metastases alone has been found to have worse prognosis compared with pulmonary metastases:^[20,32]^ the patients with extrapulmonary distant metastases in this study had mainly bone metastases.

The present study had some limitations. First, the study had a retrospective design. During the 8 years of the study period, some clinical data were missing, such as pathological subtype, angioinvasion, and positive margins, which might have caused a bias in the data selection. Second, the sample size was small, making it difficult to perform further stratified analysis for the prognostic factors. Finally, the follow-up period was short. A further study with a longer follow-up period and progression/overall survival analyses is needed, which would have more clinical significance.

## 5 Conclusion

In conclusion, our study indicated that most DTC patients with pulmonary metastases can obtain partial or complete remission after ^131^I therapy. ^131^I imaging and serum Tg levels at diagnosis are both important indicators to evaluate the curative effect. Older patients with higher Tg levels at diagnosis and extrapulmonary distant metastases more likely show poor prognosis. The optimal cutoffs for age and Tg level to predict ^131^I therapy efficacy for DTC with lung metastases were 46 years old and 55.50 ng/mL, respectively. The analysis of efficacy and prognostic factors of ^131^I therapy have the benefit to establish individualized treatment strategy, predict curative effect, and assess the prognosis for those DTC patients.

## Acknowledgments

The authors thank the National Natural Science Foundation of China grants 81501510 (awarded to RW) for the support.
